# Insects Overshoot the Expected Upslope Shift Caused by Climate Warming

**DOI:** 10.1371/journal.pone.0065842

**Published:** 2013-06-07

**Authors:** Claus Bässler, Torsten Hothorn, Roland Brandl, Jörg Müller

**Affiliations:** 1 Department of Research, Bavarian Forest National Park, Grafenau, Germany; 2 Institute for Statistics, Ludwig Maximilians University, München, Germany; 3 Department of Animal Ecology, Faculty of Biology, Philipps-Universität Marburg, Marburg, Germany; Institute of Botany, Czech Academy of Sciences, Czech Republic

## Abstract

Along elevational gradients, climate warming may lead to an upslope shift of the lower and upper range margin of organisms. A recent meta-analysis concluded that these shifts are species specific and considerably differ among taxonomic lineages. We used the opportunity to compare upper range margins of five lineages (plants, beetles, flies, hymenoptera, and birds) between 1902–1904 and 2006–2007 within one region (Bavarian Forest, Central Europe). Based on the increase in the regional mean annual temperature during this period and the regional lapse rate, the upslope shift is expected to be between 51 and 201 m. Averaged across species within lineages, the range margin of all animal lineages shifted upslope, but that of plants did not. For animals, the observed shifts were probably due to shifts in temperature and not to changes in habitat conditions. The range margin of plants is therefore apparently not constrained by temperature, a result contrasting recent findings. The mean shift of birds (165 m) was within the predicted range and consistent with a recent global meta-analysis. However, the upslope shift of the three insect lineages (>260 m) exceeded the expected shift even after considering several sources of uncertainty, which indicated a non-linear response to temperature. Our analysis demonstrated broad differences among lineages in their response to climate change even within one region. Furthermore, on the considered scale, the response of ectothermic animals was not consistent with expectations based on shifts in the mean annual temperature. Irrespective of the reasons for the overshooting of the response of the insects, these shifts lead to reorganizations in the composition of assemblages with consequences for ecosystem processes.

## Introduction

A plethora of studies have demonstrated recent shifts in the distribution of species in many marine, freshwater and terrestrial organisms [1]. Most of these shifts can be attributed to global warming [2], [3]. However, the variability of the response of species or groups of phylogenetically related species (lineages) to global warming is considerable [3]. Furthermore, the processes underlying the differences within and among lineages are not well understood, which hampers the transfer of results from one lineage to another as well as predictions of the effects of climate change on assemblages of species [3]. Two hypotheses may explain this variability among species, lineages and studies. First, species and also lineages differ in their physiological characteristics as well as traits that mediate their interaction with the environment leading to the observed differences in their response to climate change [4]. Second, the response differs among regions (e.g. because of biotic interactions that vary among assemblages differing in species composition) as well as among spatial scales (e.g. because different processes associated with climate change operate across different spatial scales). Most comparative studies, however, have to combine results from lineages investigated in different regions and/or on different geographic scales [1]. To distinguish between these two explanations, one has to compare results among species or lineages within one region. However, the availability of such data is rather limited; but see [5].

We took advantage of an opportunity to compare information from 1902–1904 and 2006–2007 on the distribution of plants, insects and birds along an elevational gradient within the low-range mountain massif of the Bavarian Forest in south-eastern Germany. Mountains are important study objects in climate change research [6]. In contrast to latitudinal studies, species are able to respond more readily to a changing climate due to the short distances along the local gradients [7], [8]. As fingerprints of climate change, contractions on the lower range margins [9], shifts of the species optimum [7] and upslope shifts of the upper range margins [10], [11] have been shown. Despite the short distances along elevational gradients, however, the observed response of species to global warming along these gradients often lag behind the response predicted from temperature shifts [2]. Furthermore, this lagging behind is not consistent across lineages [2]. The aim of our study, was to compare the average upper elevational range margins of five lineages in one region with a quantitative expectation based on climate data that were in contrast to many published studies, collected in the study area, and thereby to answer (1) whether the upper elevational range margin of each lineage in the investigated area shifts; (2) whether these shifts are consistent among lineages; and (3) whether these shifts are consistent with temperature shifts or lag behind.

## Materials and Methods

### Study area

**Figure 1:**
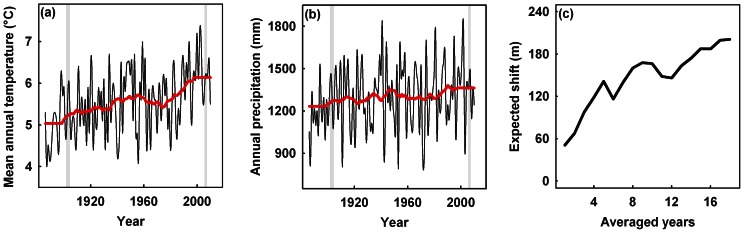
Mean annual temperature, annual precipitation and expected upslope shift over time. The first recordings of the mean annual temperature (a) and annual precipitation (b) in the study area were in 1886. The shaded areas indicate the two sampling periods (1902–1904 and 2006–2007). Each red line indicates a moving average across 20 years. Expectations in the upslope shift were calculated from the differences in temperature of the two periods and the local lapse rate (for details, see Statistical analysis).

The Bavarian Forest National Park (48°55′N, 13°28′E) lies within the largest low-range mountain forest in Central Europe, the mountain massif of the Bavarian Forest, and covers approximately 24,000 ha. It is characterized by a mountain slope increasing from south-west to north-east, which leads to a predominantly south-west exposure [12]. Elevation ranges from 650 to 1,450 m a.s.l., and Mt. Rachel is the highest mountain of the park. The high montane forest (>1,150 m) is dominated by Norway Spruce (*Picea abies*), with only a low proportion of European Beech (*Fagus sylvatica*) and Mountain Ash (*Sorbus aucuparia*); below this elevation, the mixed montane forest is dominated by spruce, beech, and Silver Fir (*Abies alba*). The temperature increased during the 20^th^ century (Fig. 1a; r^2^ = 0.19, p<0.001). The average temperature of the 20 years (1886–1905) around our first study period (1902–1904) was 5.1°C, whereas the average of the 20 years (1991–2010) around our second study period (2006–2007) was 6.2°C. Although the annual precipitation increased from 1,196 to 1,352 mm between 1886 and 2010 in the study region, the precipitation over time was more erratic (Fig. 1b; r^2^ = 0.036, p<0.05).

### Species data

From 1902 to 1904, Thiem [13] sampled several lineages of organisms along the elevational gradient of Mt. Rachel to determine their distribution and reported their upper elevational range margin. In 2006 and 2007, we surveyed two transects along Mt. Rachel similar to those of Thiem [13] as well as two transects approximately 10 km distant from Mt. Rachel in the northern part of the national park (Mt. Lackenberg). The exposure and slope of the localities of the two surveys are therefore similar. In both surveys, taxonomical experts participated in the determination of species. We harmonized the species lists with respect to synonymy and the splitting up of species. We restricted our comparison to lineages sharing at least 50 taxonomically uncritical species between the two time periods. Given this criterion, we analysed five lineages: Spermatophyta, Coleoptera, Hymenoptera (only Symphyta, Aculeata and Formicidae), Diptera (only Syrphidae) and Aves (Table 1, see also Table S1). Our own survey was carried out on fixed plots: 286 plots for Spermatophyta (relevees) and Aves (breeding birds, grid mapping), 182 plots for Coleoptera (window and pitfall traps, direct search), and 36 plots for Syrphidae and Hymenoptera (malaise traps); for methods, see [12]. All necessary permits for our field study were obtained from the local authorities (District Niederbayern).

**Table 1:** The number of species of five lineages recorded during the two sampling periods (1902–1904 and 2006–2007), the number of species shared between the two periods and the mean upper range margin of the species shared during the two periods.

Lineage	Number of species		Number of shared species	Mean upper range margin (m a.s.l.)	
	1902–1904	2006–2007		1902–1904	2006–2007
Spermatophyta	403	194	164	1,194	1,118 (1152)
Coleoptera	743	922	322	913	1,187 (1254)
Syrphidae	85	115	50	949	1,215 (1270)
Hymenoptera*	136	222	61	913	1,202 (1271)
Aves	82	76	57	1,097	1,262 (1287)

* The Hymenoptera include Symphyta, Formicidae and Aculeata.

For the period 2006–2007, the mean altitude of all species with at least two records is shown in parentheses.

### Statistical analysis

Mean annual temperature data since 1886 are available for the study region (Fig. 1). To derive an expectation for the upslope shift, we multiplied the linear lapse rate (linear decrease of temperature along elevation) of the study area (0.0059°C m^−1^), which has been shown to be robust across space and time [14], with the difference in mean annual temperature between the two survey periods. However, climatic events affect populations with a certain time lag [15]. This time lag depends on, e.g. the life span of a species (from weeks in insects to many years for some birds or plants) or the age reached at the time of the first reproduction [16]. We therefore calculated differences using an increasing number of years. We first calculated the difference in the mean annual temperature between 1903 and 2006 and added step-by-step one additional year for both the historical and the recent time series (e.g. calculating in the second step the difference between the average annual temperatures of 1901–1902 and 2005–2006) until we calculated the differences of average temperatures across 18 years (i.e. the difference between the average temperature of 1886–1903 and 1989–2006). This limit was set by the availability of data for the region (first available annual temperature record in 1886; see above and Fig. 1). Finally, we calculated the expected shifts by multiplying the differences obtained by this procedure with the lapse rate (see above). When we compared only 1903 and 2006, the expected shift was 51 m; when we used the difference of annual mean temperatures averaged across 18 years, the expected shift increased to 201 m (Fig. 1c). These two estimates were used as a conservative estimate for the interval in which the distributional shift should fall. Additional estimates based on lapse rates calculated for various seasons (e.g. the lapse rate is larger during the growing season) showed that these expected shifts also fell within the range of 51–201 m. We are aware that extreme temperatures might be more important for the biogeography of a species than mean temperature [17]. However, at present, we have no reliable data for considering such events [18].

We used the *ecdf* function (empirical cumulative distribution function) in R 2.15.0 [19], starting from the upper elevational end of the mountain to estimate the relative number of species occurring above a specific elevation [20] and using only the species shared by both surveys (Table 1). The empirical cumulative distribution function is a step function with *i/n* jumps at observation values, where *i* is the number of common observations at that elevation and *n* is the number of species. We tested differences between periods for each of the five lineages using the Kolmogorow-Smirnow test.

**Figure 2:**
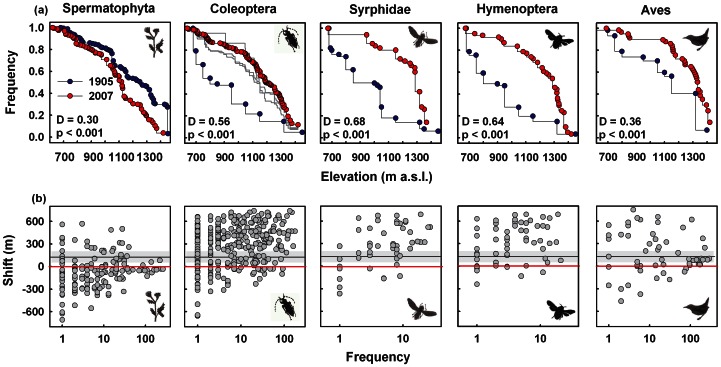
Percentage of species occurring above a specific elevation and shifts of the upper range margin of individual species in relation to frequency. (a) Comparison of the percentage of species occurring above a specific elevation in the two surveys in the low-range mountain forests of the Bavarian Forest National Park. To generate these plots, we used the empirical cumulative distribution function (for details, see Statistical methods) from 1902–1904 (blue) and 2006–2007 (red) and included only species that were recorded in both surveys. The stair-step pattern is a consequence of sampling discrete sites on the gradient. Note that the highest points sampled in each survey differ somewhat. p-values and the maximum distance (D) arising from a Kolmogorov Smirnov test are given. For Coleoptera, we recalculated the curves for species with at least 5, 10 and 50 individuals (grey lines). (b) Shift of the upper range margin between the surveys of 1902–1904 and 2006–2007 in relation to the number of plots on which a species was recorded (frequency).

We relied on the data reported by Thiem [13], which are based on the uppermost elevation record of each species. Such data depend heavily on both the sampling effort and the population size of a species, which might fluctuate considerably over years. However, we have no information on the sampling effort of the survey of Thiem [13] to allow comparison with that of our survey. Therefore, the estimates of the range shifts of single species are unreliable, and we decided to restrict ourselves on broad comparisons of the mean shift among lineages. Nevertheless, even such averages might depend on the sampling effort (see also Table 1). We therefore used three methods to estimate the possible effects of uneven sampling effort [21]. (1) During the recent survey, we recorded more species of beetles and flies than listed by Thiem [13], which indicated that our survey was more intensive [22]. We therefore recalculated the cumulative distribution of beetles (the lineages with the highest number of species; Table 1) considering only species with at least 5, 10 and 15 individuals (Fig. 2a); thus, we used only well-sampled species for our comparisons. (2) We calculated estimates of the mean shift of the upper elevational range margin, reducing the number of sampling plots in our data sets to assess the sensitivity of the difference between the two surveys to the sampling intensity of the recent survey (Fig. S1). (3) To calculate the mean shift of species within lineages, we compared the averages across all species to averages excluding species recorded only on one plot (Table 1).

## Results

The cumulative distribution of the upper elevational range margin for the two sampling periods 1902–1904 and 2006–2007 (Table 1) suggested that the elevation of the upper range margins of all five lineages differed between the two sampling periods (Fig. 2a): the mean upper range margin of plants decreased, whereas that of all animals increased. However, the apparent shift depended on the abundance and occupancy of species (Fig. 2b, Table 1). Although we have such detailed data for the second period only, a plot of the apparent range shift versus the frequency of plots with records of that species showed that the variability of the apparent shift decreases with the frequency (Fig. 2b). Nevertheless, abundant plant species showed on average a downslope shift, whereas abundant animal species showed an upslope shift (Fig. 2b; see also Table 1).

The apparent mean shift of the upper range margin of vascular plants was approximately −75 m, irrespective of the commonness of species (Fig. 2b). Thiem [13] used a correction factor to compare data of plants from different exposures. For animals, he reported no such factor. The main exposure of the plots of our recent survey was south-west, for which Thiem [13] used a correction factor of +70 m. This correction factor explains the difference between the two surveys; we therefore conclude that the upper range margins did not shift during the 20^th^ century. Overall, these analyses clearly showed shifts in the distribution of only animal species between the two periods.

**Figure 3:**
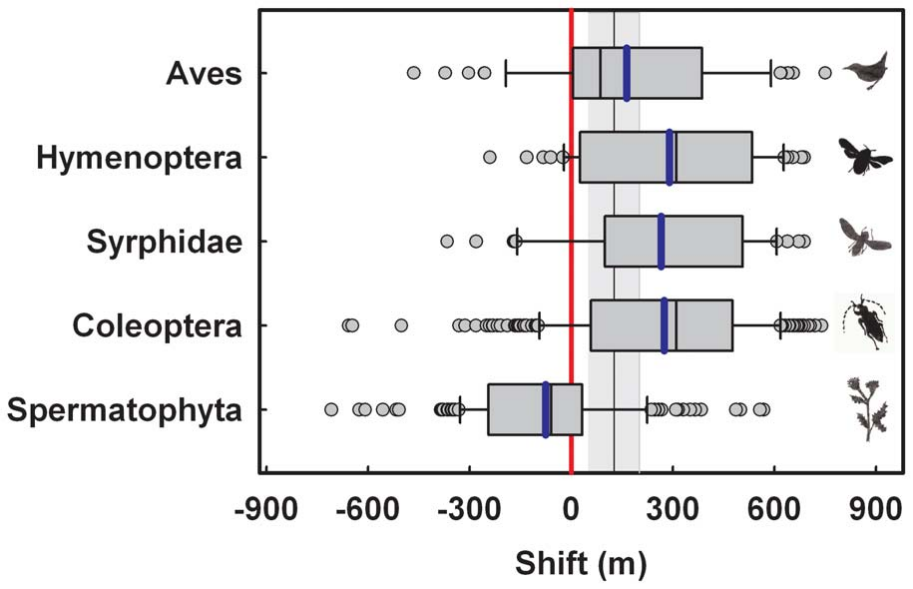
Mean shift of the upper range margin of species. Box plots of shifts in the upper ranges of single species of the lineages under study between 1902–1904 and 2006–2007. Each outlier is shown (outside the 10^th^ and 90^th^ percentiles). The mean shift of each lineage is indicated by a blue line. The black line indicates the mean expected shift of 125 m; the area shaded in grey represents the range of the expected shift of 51–201 m. Calculations are based on regional climate data (for detail, see Statistical methods).

The upslope shift of birds and insects differed considerably (Fig. 3, Table 1). The quantitative shift of the birds was as expected from the change in the mean annual temperature, but the upslope shift of all three lineages of insects clearly overshot this expectation (Fig. 3). The recalculation of the cumulative distribution of beetles considering only species with at least 5, 10 and 15 individuals changed this pattern only marginally (Fig. 2a). We reached to the same conclusion when we calculated the means after excluding species recorded only on one plot during our surveys (Table 1). Furthermore, when we reduced the number of sampling plots of the recent survey stepwise (Fig. S1), or when we concentrated on the second-highest record of each species (not shown), the differences and therefore the overshooting phenomena of the insects remained a robust outcome of our analyses. We conclude that the changes in the distributional shifts of insects and birds differed. Furthermore, only for birds was the shift within the expectation based on the temperature shift; for insects, this expectation was overshot.

## Discussion

Compared to other historical data, the survey of Thiem [13] is to our knowledge one of the most comprehensive studies documenting species distributions along an elevational gradient at that time. The data are of exceptional quality due to both the high standard of taxonomical knowledge in Central Europe and the participation of leading experts. Nevertheless, we had to consider various aspects common to all studies dealing with historical data.

A main assumption of our analysis is that the distribution of species in the region is temperature limited. This assumption is reasonable because we previously found that the composition of assemblages of numerous lineages depends on temperature [23], [24]. Furthermore, that in animals the documented shifts are probably a response to climate warming is supported by our analyses of traits of beetles. We found, consistent with the ‘thermal melanism hypothesis’ [25], a decrease in melanism in beetle assemblages with elevation between the two periods (Fig. S2).

In many studies, it is difficult to disentangle effects of climate change from effects of changes in land use or habitats [5], [26]. Clearly, land use by humans also influenced the Inner Bavarian Forest. However, the Bavarian Forest was colonized by humans relatively late during the 17^th^ century. Although timber was used in the following century for glass production, the structure of the forests changed only little from the pristine conditions. Furthermore, tree species composition of the forests was mainly triggered by natural processes and events [27]. The similar structure and composition of the two periods is particularly evident from photographs taken along the slopes of Mt. Rachel (Fig. S3). Probably because of climatic reasons [28], the forests near the summit of Mount Rachel during the first survey were as open as during our survey (Fig. S3). Furthermore, ca. 30 years before the survey of Thiem [13], the high montane spruce forest in the study area was affected by windthrow and subsequent bark beetle infestation. Dead wood was therefore abundant, as it is now [12], [29]. A final argument that habitat conditions had little influence on our broad comparisons comes from a comparison of our two transects on Mt. Rachel with the transects on Mt. Lackenberg. The latter transects are in an area with a denser canopy. However, we found no difference in the mean upper range margins among lineages (data not shown).

Most studies documenting distributional changes fail to make *a priori* predictions of the magnitude of the expected effect; but see [3]. Our analysis demonstrated that such expectations are associated with large error margins that depend on the time window used to calculate the difference between the mean annual temperatures of the two periods [2]. Nevertheless, despite this broad error margin of the expected response, the upslope shift of ectothermic insects exceeds the expected shift. In contrast, the upslope shift of birds matches the expectation calculated from the information on the regional climate. Furthermore, the observed upslope shift of birds is also consistent with a recent meta-analysis, whereas our observed shift of insects was much larger than expected from this meta-analysis [3]. Nevertheless, the response of species or lineages to climate change is difficult to predict if only the mean annual temperature is considered - extreme temperatures are more important than averages [17]. One final critical point is that we were only able to analyse two distinct time steps. We have no information about when exactly shifts in the upper range margins took place. Temperature varied considerably over time, and the major increase in temperature occurred during the last 20 years [30].

Despite the various caveats of sampling and other possible sources of errors, the careful interpretation of the available information of our study suggested that insects overshot the expected response to climate warming and plants did not respond. Generally, it is noteworthy that a lack of response or even a downward range shift does not necessarily mean that climate change has no effect. A recent study has suggested that downslope range shifts of species may constitute an indirect biotic response to both climate warming and habitat modification [31]. However, the reason for the plants' lack of response in the timeframes compared in our study became clear after a more detailed inspection of our data: the plants had reached higher elevations than insects and birds already at the beginning of the 20^th^ century (Fig. 2). The stability of the range margin of plants suggests that in contrast to animals factors other than temperature drive the elevational range margins in the region considered. This is also supported by our analysis of traits in which we found that the composition of plant assemblages based on Ellenberg indicator values did not change with altitude (Fig. S2).

The assemblages of plants on the mountain top above approximately 1,150 m a.s.l. (Calamagrostis villosae-Fagetum and -Piceetum) are characterized by a high abundance of species of the genus *Calamagrostis* [32]. Experimental evidence shows that the dominant grasses decrease the rate of establishment of other species [33]. Another genus that can also be dominant at this elevation zone is *Vaccinium*. For species of this long-lived genus, changes in the distribution owing to climate change can take decades [34]. Therefore, species of the genera *Calamagrostis* and *Vaccinium* form an effective barrier and might thus hamper the establishment of new plant species shifting uphill in response to climate change. This hypothesis has already been discussed for subalpine grasslands, where the response of plants to climate change is also weak [9]. From these findings, we can envisage that the upper range margin of most plant species in 1900 was already located in a zone in which shifting is strongly hindered by competition of perennial shrubs and grass species (most probably by root competition) [35]. This ecological situation contrasts that of the subnival/nival zone on alpine summits, where species distribution and numbers change with climate change [11], [36].

A second biome-specific explanation for the lack of the plant species response may also be that the plant assemblages of our study area on acidic soils are poor in specialists at lower and mid elevations [37]. Therefore, at lower elevations, where more signals have to be expected owing to the reduced number of perennial plants, few species show an upper distribution limit. A last argument we have to consider is the scale. In contrast to investigations of summit plant shifts conducted on the scale of meters [38], we had only coarse historical data. This leads to a masking of small-scale effects [39].

The most exciting result of our study was the considerable and robust difference in the upslope shift between birds and insects. The results for insects contrasted recent findings that the response of species along elevational gradients lags behind the expectation [3]. There are two possible explanations for this difference. First, a lag in the response is expected if suitable new conditions at higher elevations occur only in locations that cannot be reached (for example, on other mountain peaks) [3]. In our case, the spatial distance between sampling plots was maximally 10 km. Dispersal distances of insects are several kilometres per year [40], [41] and dispersal distances of plants, at least for anemochorous ones, are estimated to be in a similar range [42], which suggest that in our region, species may have reached equilibrium. Second, lags may reflect the topographic complexity of mountainous terrains leading to microclimatic mosaics with very different conditions [43]. However, this might be more relevant in alpine systems, where the topography is more complicated than along low-range mountain systems.

The question remains why birds and insects dramatically differed in their response to climate warming. One reason might be that insects react to different climatic variables (e.g. temperature during summer) than birds (e.g. temperature during spring; 54% of the bird species recorded in our area are migratory). Another possibility is based on the metabolic theory. According to this theory, temperature non-linearly affects the rates and times of ecological processes [44]. The body temperature of ectotherms depends on air temperature; therefore, ectotherms react non-linearly to changes in temperature. However, the use of lapse rates implies a linear response. In principle, it should be possible to use high-frequency temperature data of global change to predict the response of insects along elevational gradients [45]. However, our lack of knowledge of the coupling of macrophysiologial processes and processes relevant for the spatial ecology of species make such predictions difficult, although not impossible [2].

Irrespective of the reasons behind the differences in the range shift of organisms, our results showed that lineages within a region respond differently to climate change. In contrast, a meta-analysis of data from around the globe has suggested that most variability of the response is within lineages and not among lineages. Generalizations from a broad analysis obviously lead to biased results when applied to a specific region. At a minimum, our results demonstrated that the level of sensitivity to global warming differs considerably among lineages. These lineages often trigger different processes in the local assemblages and communities. Climate change therefore leads not only to reorganizations in the composition of assemblages but probably also to changes in ecosystem processes.

## Supporting Information

Figure S1 **Influence of sampling intensity on the mean shift of the upper elevational range margin of Coleoptera and of Hymenoptera and Syrphidae.**
(DOC)

Figure S2 **Traits of Coleoptera and Spermatophyta plotted across the altitudinal gradient.**
(DOC)

Figure S3 **Original photographs of the high montane spruce forest on Mt. Rachel around 1900 and today.**
(DOC)

Table S1 **Total list of shared species (n = 654) of the two surveys (1902–1904 and 2006–2007) used for the analysis and related information on the upper range margin (m a.s.l.).**
(DOC)
