# Supplementary material for: Insects Overshoot the Expected Upslope Shift Caused by Climate Warming
Source: PLoS One. 2013 Jun 7;8(6):e65842. doi: 10.1371/journal.pone.0065842 (PMC3676374; doi:10.1371/journal.pone.0065842)
Supplement: Figure S1 — Influence of sampling intensity on the mean shift of the upper elevational range margin of Coleoptera and of Hymenoptera and Syrphidae. (DOC) [file pone.0065842.s001.doc]

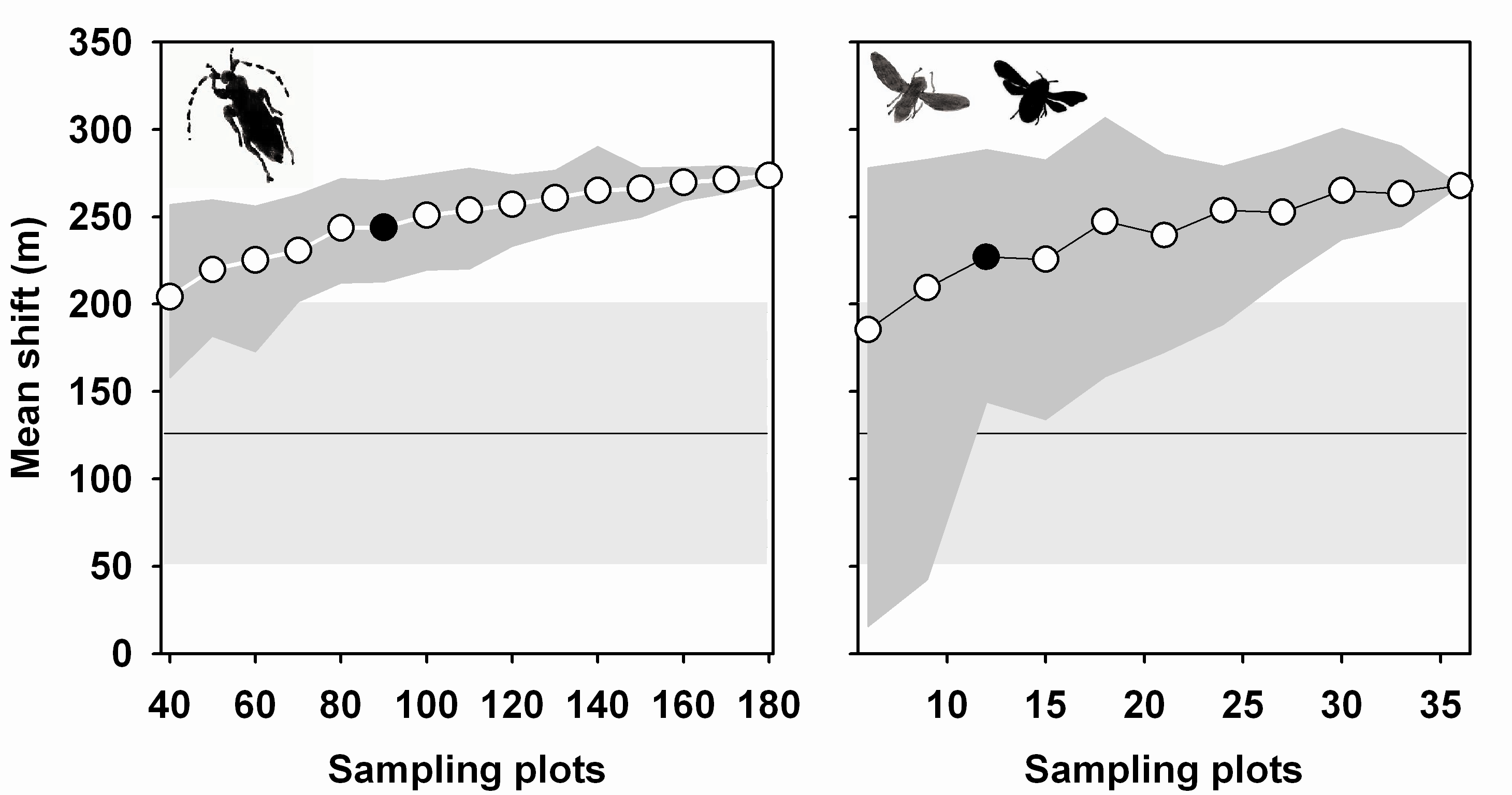


**Fig. S1:** Influence of sampling intensity on the mean shift of the upper elevational range margin of Coleoptera on 182 plots and of Hymenoptera and Syrphidae together on 36 plots. Samples were collected with malaise traps. The black point indicates the number of sample sites that leads to the same number of species in our data set as in the study of Thiem (1906) using a species–sites rarefaction curve.

day (right).
