# Supplementary material for: Insects Overshoot the Expected Upslope Shift Caused by Climate Warming
Source: PLoS One. 2013 Jun 7;8(6):e65842. doi: 10.1371/journal.pone.0065842 (PMC3676374; doi:10.1371/journal.pone.0065842)
Supplement: Figure S2 — Traits of Coleoptera and Spermatophyta plotted across the altitudinal gradient. (DOC) [file pone.0065842.s002.doc]

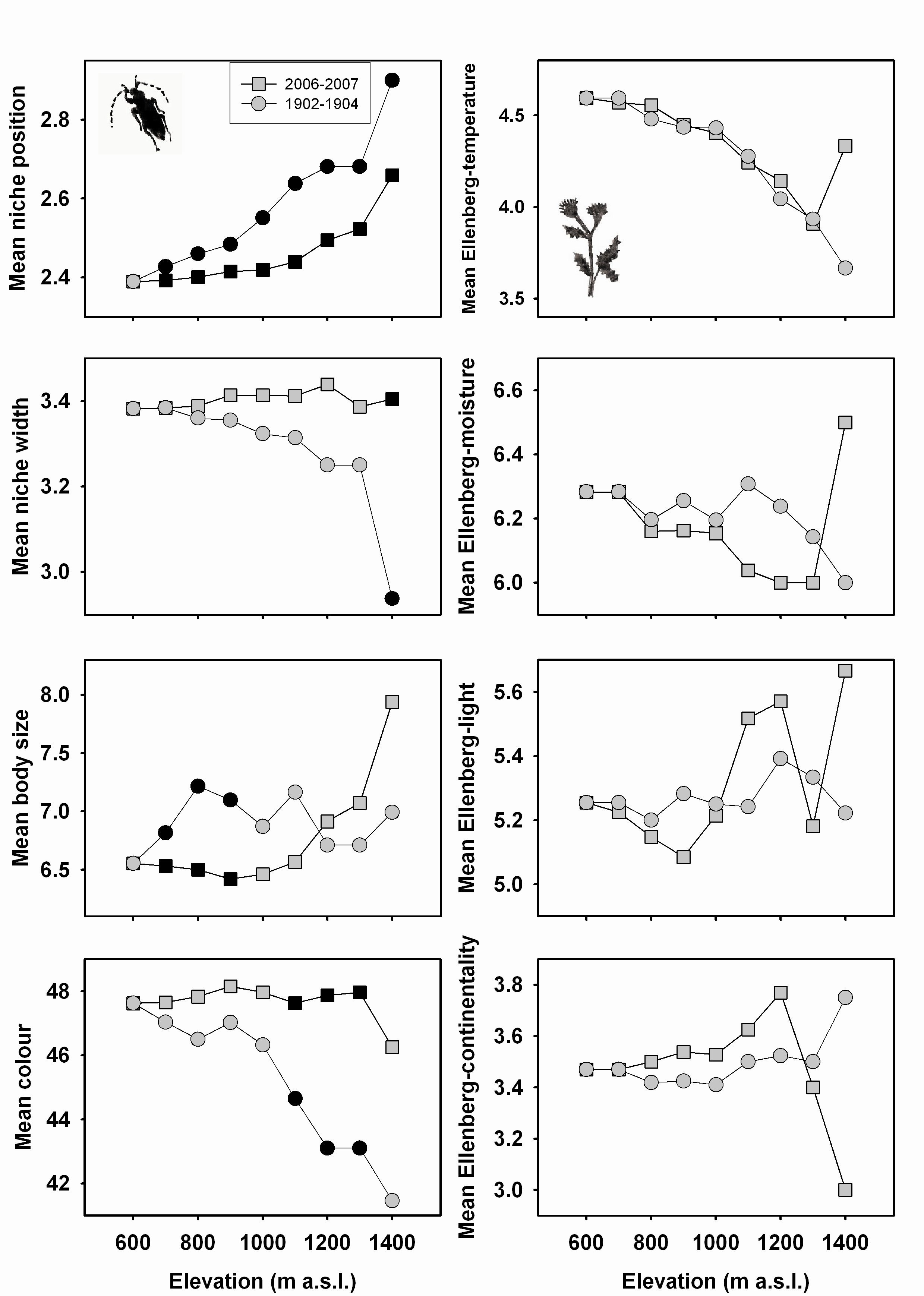


**Fig. S2:** Traits of Coleoptera (left) and Spermatophyta (right) plotted across the altitudinal gradient, cumulatively calculated as the mean in 100 m elevational steps starting at 600 m a.s.l. Filled circles, data of Thiem (1902–1904); filled squares, recent data (2006–2007). Significant differences are indicated by dark symbols. For Spermatophyta species, we used information on the Ellenberg values for temperature, moisture, light and continentality [1]. Among all shared species (164), we were able to select 59 for which all Ellenberg values were available. For Coleoptera — the most species-rich group in both surveys — we considered for all shared species (n = 322) the following traits extracted from the literature [2]: niche position (mean position across systems from planar to alpine), niche width (number of occurrences across systems from planar to alpine), and body size (mean in mm). The degree of pigmentation of each species was measured. For this, we took photographs of specimens from a regional collection, all sampled in the study area. From each colour photo, we estimated the brightness using an 8-bit grey scale with values from 0 (black) to 255 (white). Such a measurement allows a detailed pigmentation assessment even for species with pigment patches or bands in contrast to a rough classification from black to pale [3].

To test the influence of the shift on the mean trait values of the assemblages in former and recent times, we cumulatively calculated in steps of 100 m (> 599, > 699, … > 1,399) the mean trait values for both time periods, assuming that species occurred in former times in all zones below their upper range along our gradient. This assumption is strongly supported by literature in which the elevational distribution of species is provided [e.g. for Coleoptera, see 4]. To test for significant difference of the mean trait values between the two sampling periods, we randomly sampled (2,000 runs) species within the elevational steps and calculated the difference. All comparisons between the two sampling periods across the elevational steps that extend the range of the randomized values are treated as significant.

**References**

1. Ellenberg H (1991) Indicator values of plants in Central Europe 1. Indicator values of vascular plants not including Rubus. Scripta Geobotanica 18: 9-166.

2. Böhme J (2001) Phytophage Käfer und ihre Wirtspflanzen in Mitteleuropa - Ein Kompendium. Heroldsberg: Bioform. 132 p.

3. Rapoport EH (1969) Gloger's rule and pigmentation of collembola. Evolution 23: 622-626.

4. Böhme J (2005) Die Käfer Mitteleuropas: Spektrum Akademischer Verlag in Elsevier 132 p
