# Supplementary material for: Insects Overshoot the Expected Upslope Shift Caused by Climate Warming
Source: PLoS One. 2013 Jun 7;8(6):e65842. doi: 10.1371/journal.pone.0065842 (PMC3676374; doi:10.1371/journal.pone.0065842)
Supplement: Figure S3 — Original photographs of the high montane spruce forest on Mt. Rachel around 1900 and today. (DOC) [file pone.0065842.s003.doc]

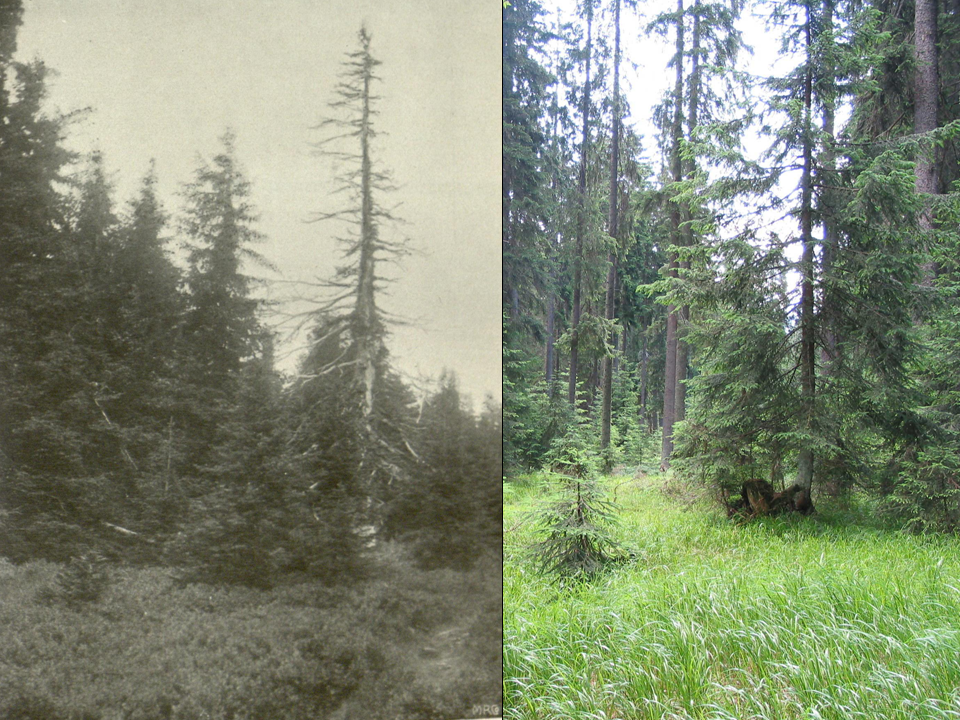


**Fig. S3:** Original photographs of the high montane spruce forest on Mt. Rachel around 1900 (left) and today (right).
