# Supplementary material for: Insects Overshoot the Expected Upslope Shift Caused by Climate Warming
Source: PLoS One. 2013 Jun 7;8(6):e65842. doi: 10.1371/journal.pone.0065842 (PMC3676374; doi:10.1371/journal.pone.0065842)
Supplement: Table S1 — Total list of shared species (n = 654) of the two surveys (1902–1904 and 2006–2007) used for the analysis and related information on the upper range margin (m a.s.l.). (DOC) [file pone.0065842.s004.doc]

Table S1: Total list of shared species (n=654, see also Table 1) of the two surveys (1902–1904 and 2006–2007) used for the analysis and related information on the upper range margin (m a.s.l.).

| Lineage | Species | Upper range margin (m a.s.l.) | |
| --- | --- | --- | --- |
|  |  | 1902-1904 | 2006-2007 |
| Spermatophyta | *Abies alba* | 1220 | 1173 |
| Spermatophyta | *Acer platanoides* | 1150 | 916 |
| Spermatophyta | *Acer pseudoplatanus* | 1320 | 1271 |
| Spermatophyta | *Achillea millefolium* | 810 | 1161 |
| Spermatophyta | *Aconitum napellus* | 1330 | 1005 |
| Spermatophyta | *Actaea spicata* | 1140 | 1007 |
| Spermatophyta | *Agrostis capillaris* | 1450 | 1287 |
| Spermatophyta | *Ajuga reptans* | 1060 | 1136 |
| Spermatophyta | *Alnus glutinosa* | 730 | 810 |
| Spermatophyta | *Andromeda polifolia* | 1250 | 742 |
| Spermatophyta | *Anemone nemorosa* | 1440 | 1108 |
| Spermatophyta | *Anthriscus sylvestris* | 1340 | 1161 |
| Spermatophyta | *Betula pendula* | 980 | 1216 |
| Spermatophyta | *Bistorta officinalis* | 1300 | 1236 |
| Spermatophyta | *Blechnum spicant* | 1320 | 1298 |
| Spermatophyta | *Calamagrostis villosa* | 1360 | 1420 |
| Spermatophyta | *Caltha palustris* | 1300 | 1323 |
| Spermatophyta | *Campanula patula* | 820 | 824 |
| Spermatophyta | *Cardamine amara* | 1140 | 944 |
| Spermatophyta | *Cardamine bulbifera* | 1110 | 803 |
| Spermatophyta | *Carex brizoides* | 1300 | 1137 |
| Spermatophyta | *Carex canescens* | 1454 | 1368 |
| Spermatophyta | *Carex echinata* | 1350 | 1380 |
| Spermatophyta | *Carex flava* | 1340 | 1045 |
| Spermatophyta | *Carex leporina* | 1450 | 1182 |
| Spermatophyta | *Carex nigra* | 1330 | 1333 |
| Spermatophyta | *Carex pallescens* | 1250 | 1090 |
| Spermatophyta | *Carex pilulifera* | 1150 | 1182 |
| Spermatophyta | *Carex remota* | 730 | 1045 |
| Spermatophyta | *Carex sylvatica* | 840 | 1045 |
| Spermatophyta | *Chaerophyllum hirsutum* | 1250 | 1136 |
| Spermatophyta | *Chrysosplenium alternifolium* | 1430 | 1045 |
| Spermatophyta | *Chrysosplenium oppositifolium* | 1140 | 786 |
| Spermatophyta | *Cicerbita alpina* | 1320 | 1136 |
| Spermatophyta | *Circaea alpina* | 1030 | 1090 |
| Spermatophyta | *Circaea intermedia* | 970 | 964 |
| Spermatophyta | *Circaea lutetiana* | 1070 | 1102 |
| Spermatophyta | *Cirsium palustre* | 1050 | 1045 |
| Spermatophyta | *Cirsium vulgare* | 1050 | 699 |
| Spermatophyta | *Corylus avellana* | 780 | 768 |
| Spermatophyta | *Crepis paludosa* | 1250 | 873 |
| Spermatophyta | *Dactylis glomerata* | 830 | 1161 |
| Spermatophyta | *Danthonia decumbens* | 1060 | 694 |
| Spermatophyta | *Deschampsia cespitosa* | 1454 | 1406 |
| Spermatophyta | *Deschampsia flexuosa* | 1454 | 1420 |
| Spermatophyta | *Dryopteris dilatata* | 1450 | 1420 |
| Spermatophyta | *Epilobium angustifolium* | 1450 | 1406 |
| Spermatophyta | *Epilobium montanum* | 1150 | 1073 |
| Spermatophyta | *Epilobium palustre* | 930 | 1137 |
| Spermatophyta | *Equisetum sylvaticum* | 1040 | 989 |
| Spermatophyta | *Eriophorum angustifolium* | 1300 | 1333 |
| Spermatophyta | *Eriophorum vaginatum* | 1320 | 1333 |
| Spermatophyta | *Fagus sylvatica* | 1230 | 1312 |
| Spermatophyta | *Festuca rubra* | 1450 | 1287 |
| Spermatophyta | *Filipendula ulmaria* | 1070 | 1045 |
| Spermatophyta | *Fragaria vesca* | 1450 | 894 |
| Spermatophyta | *Frangula alnus* | 730 | 836 |
| Spermatophyta | *Fraxinus excelsior* | 890 | 1136 |
| Spermatophyta | *Galeopsis tetrahit* | 1060 | 1154 |
| Spermatophyta | *Galium odoratum* | 1060 | 1007 |
| Spermatophyta | *Galium palustre* | 950 | 944 |
| Spermatophyta | *Galium pumilum* | 1290 | 1287 |
| Spermatophyta | *Galium rotundifolium* | 910 | 786 |
| Spermatophyta | *Gentiana pannonica* | 1440 | 1396 |
| Spermatophyta | *Geranium sylvaticum* | 1250 | 1161 |
| Spermatophyta | *Glyceria fluitans* | 1010 | 752 |
| Spermatophyta | *Gnaphalium sylvaticum* | 1450 | 1154 |
| Spermatophyta | *Gymnocarpium dryopteris* | 1320 | 1177 |
| Spermatophyta | *Hieracium lachenalii* | 1450 | 1159 |
| Spermatophyta | *Hieracium murorum* | 1450 | 1105 |
| Spermatophyta | *Hieracium pilosella* | 1450 | 1154 |
| Spermatophyta | *Holcus mollis* | 1050 | 805 |
| Spermatophyta | *Homogyne alpina* | 1450 | 1420 |
| Spermatophyta | *Huperzia selago* | 1450 | 1130 |
| Spermatophyta | *Hypericum maculatum* | 1330 | 1161 |
| Spermatophyta | *Hypochaeris radicata* | 1450 | 824 |
| Spermatophyta | *Juncus bulbosus* | 950 | 1087 |
| Spermatophyta | *Juncus effusus* | 1150 | 1287 |
| Spermatophyta | *Juncus filiformis* | 1330 | 1333 |
| Spermatophyta | *Knautia dipsacifolia* | 950 | 1045 |
| Spermatophyta | *Leontodon autumnalis* | 1450 | 1161 |
| Spermatophyta | *Ligusticum mutellina* | 1450 | 1380 |
| Spermatophyta | *Lonicera nigra* | 1200 | 909 |
| Spermatophyta | *Luzula luzuloides* | 1450 | 1396 |
| Spermatophyta | *Luzula multiflora* | 1450 | 1216 |
| Spermatophyta | *Luzula pilosa* | 1320 | 1159 |
| Spermatophyta | *Luzula sylvatica* | 1450 | 1396 |
| Spermatophyta | *Lycopodium annotinum* | 830 | 1330 |
| Spermatophyta | *Lycopodium clavatum* | 1040 | 1082 |
| Spermatophyta | *Lysimachia nemorum* | 780 | 1090 |
| Spermatophyta | *Maianthemum bifolium* | 1350 | 1368 |
| Spermatophyta | *Melampyrum pratense* | 1450 | 1406 |
| Spermatophyta | *Mercurialis perennis* | 1040 | 1007 |
| Spermatophyta | *Milium effusum* | 680 | 1045 |
| Spermatophyta | *Moehringia trinervia* | 1050 | 855 |
| Spermatophyta | *Molinia caerulea* | 1050 | 1298 |
| Spermatophyta | *Mycelis muralis* | 1320 | 1007 |
| Spermatophyta | *Myosotis scorpioides* | 1150 | 1045 |
| Spermatophyta | *Nardus stricta* | 1454 | 1380 |
| Spermatophyta | *Oreopteris limbosperma* | 1450 | 1380 |
| Spermatophyta | *Oxalis acetosella* | 1450 | 1368 |
| Spermatophyta | *Paris quadrifolia* | 1320 | 939 |
| Spermatophyta | *Petasites albus* | 1160 | 1102 |
| Spermatophyta | *Phegopteris connectilis* | 1150 | 1287 |
| Spermatophyta | *Phyteuma spicatum* | 1150 | 1007 |
| Spermatophyta | *Picea abies* | 1440 | 1406 |
| Spermatophyta | *Pimpinella major* | 830 | 690 |
| Spermatophyta | *Pinus mugo* | 1450 | 742 |
| Spermatophyta | *Plantago major* | 1050 | 1154 |
| Spermatophyta | *Poa annua* | 1454 | 1159 |
| Spermatophyta | *Poa nemoralis* | 1120 | 824 |
| Spermatophyta | *Polygonatum verticillatum* | 1352 | 1319 |
| Spermatophyta | *Populus tremula* | 1005 | 1216 |
| Spermatophyta | *Potentilla erecta* | 1350 | 1333 |
| Spermatophyta | *Prenanthes purpurea* | 1450 | 1323 |
| Spermatophyta | *Prunella vulgaris* | 820 | 1087 |
| Spermatophyta | *Quercus robur* | 700 | 656 |
| Spermatophyta | *Ranunculus acris* | 1270 | 1161 |
| Spermatophyta | *Ranunculus lanuginosus* | 1160 | 1007 |
| Spermatophyta | *Ranunculus platanifolius* | 1450 | 1136 |
| Spermatophyta | *Ranunculus repens* | 1450 | 1087 |
| Spermatophyta | *Rhinanthus minor* | 1060 | 1161 |
| Spermatophyta | *Rubus fruticosus* | 1060 | 1216 |
| Spermatophyta | *Rubus idaeus* | 1450 | 1396 |
| Spermatophyta | *Rumex acetosella* | 1450 | 1182 |
| Spermatophyta | *Rumex arifolius* | 1120 | 1333 |
| Spermatophyta | *Rumex obtusifolius* | 1220 | 1161 |
| Spermatophyta | *Sagina procumbens* | 790 | 824 |
| Spermatophyta | *Salix aurita* | 1450 | 1350 |
| Spermatophyta | *Salix caprea* | 730 | 1216 |
| Spermatophyta | *Salix cinerea* | 730 | 1298 |
| Spermatophyta | *Sambucus nigra* | 750 | 827 |
| Spermatophyta | *Sambucus racemosa* | 1050 | 1157 |
| Spermatophyta | *Sanguisorba officinalis* | 740 | 1298 |
| Spermatophyta | *Sanicula europaea* | 1020 | 965 |
| Spermatophyta | *Scirpus sylvaticus* | 1050 | 944 |
| Spermatophyta | *Scrophularia nodosa* | 1050 | 873 |
| Spermatophyta | *Senecio ovatus* | 1040 | 1298 |
| Spermatophyta | *Silene dioica* | 1450 | 1177 |
| Spermatophyta | *Silene nutans* | 1110 | 1420 |
| Spermatophyta | *Silene vulgaris* | 1450 | 1406 |
| Spermatophyta | *Soldanella montana* | 1450 | 1380 |
| Spermatophyta | *Solidago virgaurea* | 1450 | 1108 |
| Spermatophyta | *Sorbus aucuparia* | 1450 | 1406 |
| Spermatophyta | *Spergularia rubra* | 770 | 1154 |
| Spermatophyta | *Stachys sylvatica* | 820 | 1007 |
| Spermatophyta | *Stellaria alsine* | 1060 | 824 |
| Spermatophyta | *Stellaria graminea* | 1450 | 1161 |
| Spermatophyta | *Stellaria nemorum* | 1450 | 1287 |
| Spermatophyta | *Taraxacum Hamata* | 1450 | 1161 |
| Spermatophyta | *Tephroseris crispa* | 1170 | 926 |
| Spermatophyta | *Trientalis europaea* | 1450 | 1420 |
| Spermatophyta | *Trifolium repens* | 1450 | 1154 |
| Spermatophyta | *Tussilago farfara* | 750 | 824 |
| Spermatophyta | *Urtica dioica* | 1300 | 1090 |
| Spermatophyta | *Vaccinium myrtillus* | 1450 | 1406 |
| Spermatophyta | *Vaccinium oxycoccos* | 1050 | 742 |
| Spermatophyta | *Vaccinium uliginosum* | 1350 | 742 |
| Spermatophyta | *Valeriana officinalis* | 780 | 664 |
| Spermatophyta | *Veronica chamaedrys* | 1450 | 1161 |
| Spermatophyta | *Veronica montana* | 1050 | 750 |
| Spermatophyta | *Veronica officinalis* | 1350 | 1105 |
| Spermatophyta | *Viola palustris* | 1340 | 824 |
| Spermatophyta | *Viola reichenbachiana* | 1230 | 1136 |
| Coleoptera | *Scaphisoma subalpinum* | 700 | 1077 |
| Coleoptera | *Agathidium bohemicum* | 1050 | 818 |
| Coleoptera | *Agathidium dentatum* | 950 | 795 |
| Coleoptera | *Anoplotrupes stercorosus* | 1450 | 1350 |
| Coleoptera | *Geotrupes stercorarius* | 680 | 727 |
| Coleoptera | *Sphaerites glabratus* | 1450 | 1262 |
| Coleoptera | *Agathidium nigripenne* | 950 | 1102 |
| Coleoptera | *Aphodius depressus* | 800 | 1333 |
| Coleoptera | *Aphodius rufus* | 700 | 1262 |
| Coleoptera | *Pterostichus diligens* | 1320 | 1333 |
| Coleoptera | *Trechus splendens* | 1320 | 1350 |
| Coleoptera | *Trichotichnus laevicollis* | 850 | 1262 |
| Coleoptera | *Tachyta nana* | 750 | 1368 |
| Coleoptera | *Anisotoma castanea* | 1050 | 1320 |
| Coleoptera | *Pterostichus aethiops* | 1450 | 1420 |
| Coleoptera | *Harpalus latus* | 850 | 1161 |
| Coleoptera | *Tachinus pallipes* | 1050 | 1287 |
| Coleoptera | *Meloe violaceus* | 950 | 818 |
| Coleoptera | *Abax parallelus* | 700 | 803 |
| Coleoptera | *Aphodius rufipes* | 800 | 1333 |
| Coleoptera | *Ilybius quadriguttatus* | 750 | 737 |
| Coleoptera | *Aphodius granarius* | 800 | 740 |
| Coleoptera | *Crenitis punctatostriata* | 1320 | 1333 |
| Coleoptera | *Ampedus nigrinus* | 700 | 1368 |
| Coleoptera | *Bembidion mannerheimii* | 1320 | 1182 |
| Coleoptera | *Scymnus haemorrhoidalis* | 680 | 819 |
| Coleoptera | *Aphodius piceus* | 1320 | 1380 |
| Coleoptera | *Pterostichus melanarius* | 1320 | 1161 |
| Coleoptera | *Amara communis* | 680 | 1161 |
| Coleoptera | *Pterostichus pumilio* | 1320 | 1420 |
| Coleoptera | *Molops elatus* | 750 | 945 |
| Coleoptera | *Aphodius fimetarius* | 1320 | 1102 |
| Coleoptera | *Mordella aculeata* | 680 | 800 |
| Coleoptera | *Phaedon cochleariae* | 950 | 800 |
| Coleoptera | *Harpalus laevipes* | 950 | 1216 |
| Coleoptera | *Cryptocephalus labiatus* | 1150 | 819 |
| Coleoptera | *Mycetoporus solidicornis* | 680 | 740 |
| Coleoptera | *Calathus micropterus* | 1320 | 1406 |
| Coleoptera | *Tillus elongatus* | 950 | 1090 |
| Coleoptera | *Pterostichus niger* | 800 | 1216 |
| Coleoptera | *Quedius fuliginosus* | 680 | 1380 |
| Coleoptera | *Pterostichus nigrita* | 750 | 1252 |
| Coleoptera | *Bembidion lampros* | 700 | 1182 |
| Coleoptera | *Asiorestia femorata* | 1050 | 1333 |
| Coleoptera | *Ampedus aethiops* | 1320 | 1380 |
| Coleoptera | *Quedius alpestris* | 1450 | 1420 |
| Coleoptera | *Bembidion quadrimaculatum* | 680 | 1262 |
| Coleoptera | *Amara familiaris* | 700 | 1154 |
| Coleoptera | *Acrotrichis sericans* | 750 | 987 |
| Coleoptera | *Quedius punctatellus* | 1450 | 1420 |
| Coleoptera | *Pterostichus burmeisteri* | 1150 | 1334 |
| Coleoptera | *Platystethus arenarius* | 1150 | 1182 |
| Coleoptera | *Harpalus affinis* | 680 | 798 |
| Coleoptera | *Altica oleracea* | 680 | 1252 |
| Coleoptera | *Sinodendron cylindricum* | 1050 | 1108 |
| Coleoptera | *Amara lunicollis* | 1050 | 1368 |
| Coleoptera | *Anotylus tetracarinatus* | 700 | 1319 |
| Coleoptera | *Pterostichus oblongopunctatus* | 950 | 1368 |
| Coleoptera | *Cryptopleurum minutum* | 680 | 1247 |
| Coleoptera | *Anotylus rugosus* | 700 | 1319 |
| Coleoptera | *Lathrobium fulvipenne* | 1450 | 1406 |
| Coleoptera | *Rhyncolus ater* | 750 | 1420 |
| Coleoptera | *Cis boleti* | 1050 | 1157 |
| Coleoptera | *Bolitophagus reticulatus* | 950 | 1203 |
| Coleoptera | *Proteinus brachypterus* | 700 | 1380 |
| Coleoptera | *Bibloporus bicolor* | 750 | 1184 |
| Coleoptera | *Crypturgus pusillus* | 680 | 1420 |
| Coleoptera | *Triplax aenea* | 1150 | 900 |
| Coleoptera | *Agabus bipustulatus* | 700 | 737 |
| Coleoptera | *Philonthus carbonarius* | 680 | 795 |
| Coleoptera | *Phyllotreta tetrastigma* | 700 | 800 |
| Coleoptera | *Cychrus attenuatus* | 750 | 1420 |
| Coleoptera | *Byrrhus glabratus* | 950 | 1420 |
| Coleoptera | *Agonum sexpunctatum* | 680 | 1340 |
| Coleoptera | *Cis glabratus* | 750 | 1139 |
| Coleoptera | *Tachyporus chrysomelinus* | 680 | 1182 |
| Coleoptera | *Quedius mesomelinus* | 950 | 1236 |
| Coleoptera | *Lordithon trimaculatus* | 750 | 929 |
| Coleoptera | *Abax parallelepipedus* | 950 | 1296 |
| Coleoptera | *Megasternum obscurum* | 1320 | 1380 |
| Coleoptera | *Tachyerges stigma* | 950 | 810 |
| Coleoptera | *Philonthus cognatus* | 800 | 1020 |
| Coleoptera | *Hypnoidus riparius* | 1320 | 1182 |
| Coleoptera | *Bryaxis nodicornis* | 680 | 926 |
| Coleoptera | *Cis jacquemartii* | 750 | 1128 |
| Coleoptera | *Notiophilus biguttatus* | 1450 | 1220 |
| Coleoptera | *Dasytes fusculus* | 950 | 834 |
| Coleoptera | *Protapion fulvipes* | 680 | 1060 |
| Coleoptera | *Omalium rivulare* | 950 | 1380 |
| Coleoptera | *Tachinus laticollis* | 1050 | 1352 |
| Coleoptera | *Anisotoma humeralis* | 800 | 1320 |
| Coleoptera | *Cychrus caraboides* | 750 | 1262 |
| Coleoptera | *Otiorhynchus morio* | 1320 | 1007 |
| Coleoptera | *Anaspis rufilabris* | 700 | 1420 |
| Coleoptera | *Quedius cinctus* | 700 | 1420 |
| Coleoptera | *Domene scabricollis* | 1150 | 1420 |
| Coleoptera | *Galeruca tanaceti* | 950 | 1161 |
| Coleoptera | *Agabus melanarius* | 1150 | 1323 |
| Coleoptera | *Aleochara lanuginosa* | 750 | 1105 |
| Coleoptera | *Cis nitidus* | 680 | 1090 |
| Coleoptera | *Acupalpus flavicollis* | 750 | 1108 |
| Coleoptera | *Ostoma ferruginea* | 1320 | 1396 |
| Coleoptera | *Loricera pilicornis* | 1050 | 1106 |
| Coleoptera | *Cyphon coarctatus* | 800 | 1261 |
| Coleoptera | *Meligethes denticulatus* | 950 | 1350 |
| Coleoptera | *Otiorhynchus fuscipes* | 1150 | 1396 |
| Coleoptera | *Cis dentatus* | 1050 | 1304 |
| Coleoptera | *Hemicrepidius niger* | 1050 | 1203 |
| Coleoptera | *Rugilus rufipes* | 680 | 1090 |
| Coleoptera | *Dasytes niger* | 700 | 1406 |
| Coleoptera | *Calathus melanocephalus* | 1320 | 1161 |
| Coleoptera | *Staphylinus fossor* | 680 | 847 |
| Coleoptera | *Tachyporus ruficollis* | 1320 | 1323 |
| Coleoptera | *Magdalis nitida* | 680 | 1352 |
| Coleoptera | *Carabus glabratus* | 1150 | 1368 |
| Coleoptera | *Ancistronycha cyanipennis* | 800 | 1108 |
| Coleoptera | *Eusphalerum florale* | 950 | 1380 |
| Coleoptera | *Luperus viridipennis* | 800 | 1150 |
| Coleoptera | *Notothecta flavipes* | 950 | 669 |
| Coleoptera | *Aphodius prodromus* | 950 | 1262 |
| Coleoptera | *Lordithon exoletus* | 1150 | 1163 |
| Coleoptera | *Quedius scintillans* | 680 | 1157 |
| Coleoptera | *Cerylon histeroides* | 700 | 810 |
| Coleoptera | *Tachinus proximus* | 750 | 1284 |
| Coleoptera | *Anoplus plantaris* | 680 | 1334 |
| Coleoptera | *Monotoma longicollis* | 680 | 1225 |
| Coleoptera | *Athous zebei* | 950 | 1420 |
| Coleoptera | *Latridius minutus* | 680 | 715 |
| Coleoptera | *Atheta laevana* | 750 | 1163 |
| Coleoptera | *Necrophorus vespilloides* | 950 | 1420 |
| Coleoptera | *Stenus bifoveolatus* | 700 | 1182 |
| Coleoptera | *Acrotrichis intermedia* | 1150 | 1287 |
| Coleoptera | *Malthodes guttifer* | 950 | 945 |
| Coleoptera | *Thymalus limbatus* | 950 | 1420 |
| Coleoptera | *Tachyporus hypnorum* | 680 | 1284 |
| Coleoptera | *Rhizophagus dispar* | 1050 | 1420 |
| Coleoptera | *Hylobius piceus* | 1320 | 1420 |
| Coleoptera | *Anthonomus rubi* | 700 | 1141 |
| Coleoptera | *Otiorhynchus niger* | 1450 | 1396 |
| Coleoptera | *Pityokteines curvidens* | 1050 | 1073 |
| Coleoptera | *Taphrorychus bicolor* | 1050 | 1090 |
| Coleoptera | *Rugilus erichsoni* | 680 | 1177 |
| Coleoptera | *Carabus convexus* | 750 | 674 |
| Coleoptera | *Anthophagus omalinus* | 1450 | 1420 |
| Coleoptera | *Anthophagus alpestris* | 1450 | 1420 |
| Coleoptera | *Meligethes viridescens* | 1320 | 1284 |
| Coleoptera | *Protapion apricans* | 680 | 909 |
| Coleoptera | *Cis castaneus* | 1050 | 1312 |
| Coleoptera | *Philonthus varians* | 700 | 1333 |
| Coleoptera | *Cychramus variegatus* | 700 | 1318 |
| Coleoptera | *Oxyporus maxillosus* | 750 | 812 |
| Coleoptera | *Oiceoptoma thoracica* | 750 | 1350 |
| Coleoptera | *Glocianus punctiger* | 680 | 679 |
| Coleoptera | *Corticarina fuscula* | 800 | 1306 |
| Coleoptera | *Callidium violaceum* | 680 | 1226 |
| Coleoptera | *Scymnus abietis* | 1320 | 1368 |
| Coleoptera | *Dryocoetes autographus* | 950 | 1420 |
| Coleoptera | *Hylobius abietis* | 800 | 1380 |
| Coleoptera | *Longitarsus luridus* | 680 | 810 |
| Coleoptera | *Phloeonomus pusillus* | 950 | 1060 |
| Coleoptera | *Phyllopertha horticola* | 950 | 1368 |
| Coleoptera | *Stenus impressus* | 680 | 1334 |
| Coleoptera | *Anaspis frontalis* | 950 | 1420 |
| Coleoptera | *Eusphalerum alpinum* | 700 | 1007 |
| Coleoptera | *Agonum muelleri* | 680 | 1080 |
| Coleoptera | *Epuraea pygmaea* | 700 | 1420 |
| Coleoptera | *Carabus violaceus* | 1450 | 1352 |
| Coleoptera | *Liogluta alpestris* | 1320 | 1154 |
| Coleoptera | *Atheta fungi* | 1320 | 1219 |
| Coleoptera | *Atheta coriaria* | 950 | 742 |
| Coleoptera | *Sericus brunneus* | 750 | 1368 |
| Coleoptera | *Hydroporus palustris* | 700 | 737 |
| Coleoptera | *Carabus silvestris* | 1150 | 1420 |
| Coleoptera | *Omalium caesum* | 950 | 1420 |
| Coleoptera | *Thanatophilus rugosus* | 750 | 1219 |
| Coleoptera | *Dromius agilis* | 680 | 1323 |
| Coleoptera | *Pityogenes chalcographus* | 1450 | 1420 |
| Coleoptera | *Atheta crassicornis* | 950 | 1320 |
| Coleoptera | *Phyllotreta undulata* | 680 | 1368 |
| Coleoptera | *Rhynchaenus fagi* | 1450 | 1287 |
| Coleoptera | *Atheta picipes* | 1450 | 793 |
| Coleoptera | *Ceutorhynchus erysimi* | 750 | 1182 |
| Coleoptera | *Atheta myrmecobia* | 800 | 1182 |
| Coleoptera | *Necrophorus vespillo* | 950 | 990 |
| Coleoptera | *Acrotrichis thoracica* | 750 | 1108 |
| Coleoptera | *Lordithon lunulatus* | 1150 | 1287 |
| Coleoptera | *Ocypus aeneocephalus* | 680 | 1161 |
| Coleoptera | *Ampedus sanguineus* | 680 | 723 |
| Coleoptera | *Atomaria nigriventris* | 680 | 1108 |
| Coleoptera | *Pityophagus ferrugineus* | 700 | 1286 |
| Coleoptera | *Epuraea neglecta* | 750 | 1060 |
| Coleoptera | *Eusphalerum limbatum* | 950 | 1396 |
| Coleoptera | *Gabrius splendidulus* | 750 | 752 |
| Coleoptera | *Pissodes piceae* | 950 | 1197 |
| Coleoptera | *Athous vittatus* | 950 | 1090 |
| Coleoptera | *Philonthus fimetarius* | 680 | 909 |
| Coleoptera | *Megarthrus sinuatocollis* | 680 | 1080 |
| Coleoptera | *Atheta gagatina* | 680 | 964 |
| Coleoptera | *Anthobium melanocephalum* | 950 | 1320 |
| Coleoptera | *Cerylon fagi* | 1050 | 944 |
| Coleoptera | *Gyrophaena boleti* | 750 | 1368 |
| Coleoptera | *Melanotus castanipes* | 950 | 1420 |
| Coleoptera | *Gaurotes virginea* | 750 | 1330 |
| Coleoptera | *Cryptophagus pubescens* | 680 | 1141 |
| Coleoptera | *Cryptophagus scanicus* | 1150 | 1396 |
| Coleoptera | *Limodromus assimilis* | 1050 | 810 |
| Coleoptera | *Ips typographus* | 1150 | 1420 |
| Coleoptera | *Corymbia rubra* | 750 | 1340 |
| Coleoptera | *Elaphrus cupreus* | 680 | 737 |
| Coleoptera | *Anthaxia quadripunctata* | 1320 | 1286 |
| Coleoptera | *Hylurgops palliatus* | 700 | 1284 |
| Coleoptera | *Phyllobius calcaratus* | 700 | 1182 |
| Coleoptera | *Elodes marginata* | 800 | 1007 |
| Coleoptera | *Ctenicera cuprea* | 1050 | 1368 |
| Coleoptera | *Thanasimus formicarius* | 950 | 1420 |
| Coleoptera | *Stenurella melanura* | 850 | 1340 |
| Coleoptera | *Amischa analis* | 1320 | 1350 |
| Coleoptera | *Cratosilis denticollis* | 1320 | 1380 |
| Coleoptera | *Acalles camelus* | 680 | 1157 |
| Coleoptera | *Stenus clavicornis* | 680 | 1154 |
| Coleoptera | *Atheta sodalis* | 950 | 1368 |
| Coleoptera | *Hylastes angustatus* | 950 | 1214 |
| Coleoptera | *Phloeostiba planus* | 950 | 929 |
| Coleoptera | *Dasytes plumbeus* | 700 | 1323 |
| Coleoptera | *Eusphalerum stramineum* | 700 | 1420 |
| Coleoptera | *Carabus auronitens* | 1320 | 1420 |
| Coleoptera | *Corymbia maculicornis* | 800 | 936 |
| Coleoptera | *Cantharis obscura* | 1320 | 674 |
| Coleoptera | *Serica brunna* | 800 | 779 |
| Coleoptera | *Othius myrmecophilus* | 1150 | 1312 |
| Coleoptera | *Agrypnus murina* | 950 | 834 |
| Coleoptera | *Trichius fasciatus* | 950 | 1177 |
| Coleoptera | *Pteryngium crenatum* | 1050 | 1368 |
| Coleoptera | *Cerylon ferrugineum* | 750 | 1090 |
| Coleoptera | *Phyllotreta nemorum* | 1320 | 1368 |
| Coleoptera | *Nudobius lentus* | 800 | 1420 |
| Coleoptera | *Micropeplus porcatus* | 680 | 1136 |
| Coleoptera | *Plectophloeus fischeri* | 750 | 1215 |
| Coleoptera | *Sitona hispidulus* | 1320 | 819 |
| Coleoptera | *Hallomenus binotatus* | 700 | 1380 |
| Coleoptera | *Eusphalerum longipenne* | 700 | 1368 |
| Coleoptera | *Athous subfuscus* | 950 | 1420 |
| Coleoptera | *Chrysolina fastuosa* | 1150 | 1090 |
| Coleoptera | *Catops tristis* | 1050 | 1420 |
| Coleoptera | *Dinaraea angustula* | 1320 | 1007 |
| Coleoptera | *Agrilus viridis* | 1050 | 1090 |
| Coleoptera | *Coccinella septempunctata* | 1450 | 1368 |
| Coleoptera | *Byrrhus pilula* | 750 | 1253 |
| Coleoptera | *Gyrophaena angustata* | 700 | 1157 |
| Coleoptera | *Otiorhynchus scaber* | 1050 | 1108 |
| Coleoptera | *Cychramus luteus* | 700 | 1215 |
| Coleoptera | *Anobium pertinax* | 680 | 1203 |
| Coleoptera | *Ischnopterapion virens* | 680 | 1350 |
| Coleoptera | *Ptinus fur* | 680 | 740 |
| Coleoptera | *Rhagium bifasciatum* | 750 | 1420 |
| Coleoptera | *Alosterna tabacicolor* | 700 | 1084 |
| Coleoptera | *Molorchus minor* | 1150 | 1352 |
| Coleoptera | *Atheta pallidicornis* | 680 | 800 |
| Coleoptera | *Aphidecta obliterata* | 680 | 1334 |
| Coleoptera | *Cantharis pagana* | 950 | 1284 |
| Coleoptera | *Atheta longicornis* | 750 | 1216 |
| Coleoptera | *Cryptophagus dentatus* | 680 | 1247 |
| Coleoptera | *Oxymirus cursor* | 950 | 1420 |
| Coleoptera | *Amphichroum canaliculatum* | 1150 | 1420 |
| Coleoptera | *Cyphon variabilis* | 700 | 1081 |
| Coleoptera | *Xyloterus lineatus* | 950 | 1420 |
| Coleoptera | *Acidota cruentata* | 1050 | 1406 |
| Coleoptera | *Rhagium mordax* | 750 | 1368 |
| Coleoptera | *Evodinus clathratus* | 950 | 1340 |
| Coleoptera | *Epuraea variegata* | 950 | 1215 |
| Coleoptera | *Anastrangalia sanguinolenta* | 680 | 1350 |
| Coleoptera | *Otiorhynchus subdentatus* | 950 | 1352 |
| Coleoptera | *Xyloterus domesticus* | 680 | 1184 |
| Coleoptera | *Sitona sulcifrons* | 1320 | 1368 |
| Coleoptera | *Monochamus sutor* | 1150 | 1215 |
| Coleoptera | *Cantharis pellucida* | 1050 | 1007 |
| Coleoptera | *Pyropterus nigroruber* | 700 | 1287 |
| Coleoptera | *Sciodrepoides watsoni* | 680 | 1420 |
| Coleoptera | *Dalopius marginatus* | 750 | 1396 |
| Coleoptera | *Ischnomera sanguinicollis* | 700 | 1047 |
| Coleoptera | *Geostiba circellaris* | 750 | 1182 |
| Coleoptera | *Neogalerucella lineola* | 680 | 1334 |
| Coleoptera | *Arhopalus rusticus* | 680 | 827 |
| Coleoptera | *Oxypoda alternans* | 1150 | 1380 |
| Coleoptera | *Hylecoetus dermestoides* | 950 | 1396 |
| Coleoptera | *Phyllobius pyri* | 700 | 1177 |
| Coleoptera | *Polydrusus impar* | 680 | 994 |
| Coleoptera | *Schizotus pectinicornis* | 1150 | 1137 |
| Coleoptera | *Anthribus albinus* | 680 | 1287 |
| Coleoptera | *Dasytes cyaneus* | 950 | 847 |
| Coleoptera | *Polydrusus amoenus* | 1450 | 1396 |
| Coleoptera | *Rhagonycha lignosa* | 950 | 1215 |
| Coleoptera | *Haploglossa villosula* | 1150 | 1220 |
| Coleoptera | *Carabus linnei* | 800 | 1396 |
| Coleoptera | *Polydrusus pallidus* | 680 | 1352 |
| Coleoptera | *Propylea quatuordecimpunctata* | 700 | 1287 |
| Coleoptera | *Cantharis nigricans* | 950 | 1182 |
| Coleoptera | *Oxypoda annularis* | 750 | 944 |
| Coleoptera | *Rhagonycha testacea* | 950 | 1150 |
| Coleoptera | *Sitona puncticollis* | 1320 | 819 |
| Coleoptera | *Epuraea terminalis* | 680 | 1007 |
| Coleoptera | *Strophosoma melanogrammum* | 750 | 1312 |
| Coleoptera | *Lagria hirta* | 680 | 1080 |
| Coleoptera | *Polydrusus undatus* | 1050 | 1323 |
| Coleoptera | *Podabrus alpinus* | 1050 | 1352 |
| Coleoptera | *Asiorestia ferruginea* | 680 | 1154 |
| Coleoptera | *Hypera rumicis* | 680 | 1080 |
| Coleoptera | *Anatis ocellata* | 1050 | 1368 |
| Coleoptera | *Phyllobius arborator* | 700 | 1287 |
| Coleoptera | *Epuraea longula* | 680 | 750 |
| Coleoptera | *Absidia schoenherri* | 1320 | 1368 |
| Coleoptera | *Byturus tomentosus* | 750 | 1334 |
| Coleoptera | *Calvia quatuordecimguttata* | 680 | 1150 |
| Coleoptera | *Pachytodes cerambyciformis* | 680 | 1007 |
| Coleoptera | *Cantharis livida* | 950 | 944 |
| Coleoptera | *Stephostethus angusticollis* | 680 | 727 |
| Coleoptera | *Simo hirticornis* | 700 | 964 |
| Coleoptera | *Lochmaea capreae* | 700 | 1215 |
| Coleoptera | *Dictyopterus aurora* | 1050 | 1420 |
| Coleoptera | *Pidonia lurida* | 750 | 1137 |
| Coleoptera | *Epuraea aestiva* | 1050 | 1380 |
| Coleoptera | *Phyllobius argentatus* | 950 | 1334 |
| Syrphidae | *Baccha elongata* | 1040 | 1334 |
| Syrphidae | *Brachypalpus laphriformis* | 700 | 1287 |
| Syrphidae | *Cheilosia albitarsis* | 850 | 1150 |
| Syrphidae | *Cheilosia pagana* | 680 | 674 |
| Syrphidae | *Cheilosia variabilis* | 800 | 945 |
| Syrphidae | *Chrysotoxum arcuatum* | 1050 | 1287 |
| Syrphidae | *Chrysotoxum bicinctum* | 680 | 674 |
| Syrphidae | *Chrysotoxum fasciolatum* | 690 | 1287 |
| Syrphidae | *Criorhina asilica* | 1040 | 1007 |
| Syrphidae | *Dasysyrphus hilaris* | 1020 | 1287 |
| Syrphidae | *Dasysyrphus pinastri* | 1050 | 1319 |
| Syrphidae | *Dasysyrphus venustus* | 800 | 1252 |
| Syrphidae | *Didea fasciata* | 1040 | 674 |
| Syrphidae | *Episyrphus balteatus* | 800 | 1319 |
| Syrphidae | *Eristalis pertinax* | 850 | 1287 |
| Syrphidae | *Eristalis rupium* | 850 | 945 |
| Syrphidae | *Eristalis tenax* | 1450 | 1287 |
| Syrphidae | *Eupeodes corollae* | 750 | 1352 |
| Syrphidae | *Eupeodes luniger* | 1150 | 1319 |
| Syrphidae | *Helophilus pendulus* | 680 | 1319 |
| Syrphidae | *Helophilus trivittatus* | 680 | 945 |
| Syrphidae | *Ischyrosyrphus glaucius* | 1040 | 1150 |
| Syrphidae | *Leucozona lucorum* | 1450 | 1284 |
| Syrphidae | *Megasyrphus erraticus* | 1320 | 1287 |
| Syrphidae | *Melangyna umbellatarum* | 680 | 1352 |
| Syrphidae | *Melanogaster nuda* | 680 | 1287 |
| Syrphidae | *Melanostoma mellinum* | 1050 | 1368 |
| Syrphidae | *Melanostoma scalare* | 1000 | 1319 |
| Syrphidae | *Meliscaeva auricollis* | 1040 | 1352 |
| Syrphidae | *Meliscaeva cinctella* | 680 | 1368 |
| Syrphidae | *Myathropa florea* | 680 | 1352 |
| Syrphidae | *Neoascia meticulosa* | 680 | 945 |
| Syrphidae | *Parasyrphus lineola* | 800 | 1368 |
| Syrphidae | *Parasyrphus macularis* | 850 | 1352 |
| Syrphidae | *Pipiza noctiluca* | 1040 | 1215 |
| Syrphidae | *Pipiza quadrimaculata* | 1050 | 1150 |
| Syrphidae | *Platycheirus albimanus* | 850 | 1368 |
| Syrphidae | *Platycheirus clypeatus* | 800 | 1368 |
| Syrphidae | *Platycheirus peltatus* | 1320 | 1150 |
| Syrphidae | *Rhingia campestris* | 1320 | 1039 |
| Syrphidae | *Scaeva pyrastri* | 800 | 987 |
| Syrphidae | *Sericomyia lappona* | 1450 | 1319 |
| Syrphidae | *Sericomyia silentis* | 1040 | 1319 |
| Syrphidae | *Sphaerophoria scripta* | 1350 | 1368 |
| Syrphidae | *Sphegina clunipes* | 1050 | 1284 |
| Syrphidae | *Sphegina spheginea* | 700 | 1182 |
| Syrphidae | *Syrphus ribesii* | 1040 | 1368 |
| Syrphidae | *Syrphus torvus* | 1150 | 1287 |
| Syrphidae | *Volucella pellucens* | 1040 | 1261 |
| Syrphidae | *Xylota sylvarum* | 850 | 1352 |
| Hymenoptera | *Andrena ovatula* | 680 | 674 |
| Hymenoptera | *Ectemnius cephalotes* | 680 | 1261 |
| Hymenoptera | *Vespula vulgaris* | 680 | 1215 |
| Hymenoptera | *Andrena wilkella* | 700 | 1380 |
| Hymenoptera | *Andrena humilis* | 750 | 1245 |
| Hymenoptera | *Argogorytes mystaceus* | 750 | 1039 |
| Hymenoptera | *Bombus hypnorum* | 750 | 1334 |
| Hymenoptera | *Bombus wurfleini* | 750 | 1318 |
| Hymenoptera | *Chrysis ignita form A* | 750 | 1368 |
| Hymenoptera | *Dolichovespula saxonica* | 750 | 1323 |
| Hymenoptera | *Trichrysis cyanea* | 750 | 1225 |
| Hymenoptera | *Trypoxylon figulus* | 750 | 1287 |
| Hymenoptera | *Auplopus carbonarius* | 800 | 795 |
| Hymenoptera | *Mellinus arvensis* | 800 | 945 |
| Hymenoptera | *Bombus pascuorum* | 900 | 1368 |
| Hymenoptera | *Symmorphus allobrogus* | 1050 | 1039 |
| Hymenoptera | *Vespula rufa* | 1050 | 1368 |
| Hymenoptera | *Dolichovespula norwegica* | 1150 | 1380 |
| Hymenoptera | *Dolichovespula sylvestris* | 1150 | 1318 |
| Hymenoptera | *Andrena lapponica* | 1320 | 1420 |
| Hymenoptera | *Bombus pratorum* | 1320 | 1396 |
| Hymenoptera | *Bombus rupestris* | 1320 | 1368 |
| Hymenoptera | *Dolerus aeneus* | 1450 | 1368 |
| Hymenoptera | *Pristiphora mollis* | 1450 | 1319 |
| Hymenoptera | *Strongylogaster mixta* | 1320 | 1368 |
| Hymenoptera | *Caliroa annulipes* | 1320 | 1080 |
| Hymenoptera | *Tenthredo mesomela* | 1150 | 1150 |
| Hymenoptera | *Claremontia alternipes* | 1050 | 1215 |
| Hymenoptera | *Pachynematus scutellatus* | 1050 | 1352 |
| Hymenoptera | *Strongylogaster macula* | 1050 | 1420 |
| Hymenoptera | *Dolerus nigratus* | 950 | 1182 |
| Hymenoptera | *Aglaostigma fulvipes* | 950 | 926 |
| Hymenoptera | *Gilpinia hercyniae* | 950 | 1304 |
| Hymenoptera | *Pachyprotasis rapae* | 950 | 1287 |
| Hymenoptera | *Birka cinereipes* | 950 | 1287 |
| Hymenoptera | *Rhogogaster viridis* | 950 | 1039 |
| Hymenoptera | *Dolerus vestigialis* | 950 | 945 |
| Hymenoptera | *Tenthredo colon* | 950 | 1261 |
| Hymenoptera | *Macrophya duodecimpunctata* | 950 | 1182 |
| Hymenoptera | *Macrophya alboannulata* | 950 | 926 |
| Hymenoptera | *Tenthredo atra* | 800 | 1150 |
| Hymenoptera | *Tenthredopsis nassata* | 800 | 1334 |
| Hymenoptera | *Tenthredo livida* | 800 | 1319 |
| Hymenoptera | *Xeris spectrum* | 750 | 909 |
| Hymenoptera | *Tenthredo arcuata* | 750 | 1380 |
| Hymenoptera | *Dolerus gonager* | 680 | 1368 |
| Hymenoptera | *Tenthredo olivacea* | 680 | 1334 |
| Hymenoptera | *Eutomostethus luteiventris* | 680 | 1287 |
| Hymenoptera | *Pamphilius gyllenhali* | 680 | 674 |
| Hymenoptera | *Dolerus picipes* | 680 | 1182 |
| Hymenoptera | *Tenthredo temula* | 680 | 674 |
| Hymenoptera | *Athalia cordata* | 680 | 1287 |
| Hymenoptera | *Athalia circularis* | 680 | 1368 |
| Hymenoptera | *Taxonus agrorum* | 680 | 1319 |
| Hymenoptera | *Ametastegia pallipes* | 680 | 1137 |
| Hymenoptera | *Manica rubida* | 700 | 1105 |
| Hymenoptera | *Lasius mixtus* | 800 | 740 |
| Hymenoptera | *Formica sanguinea* | 950 | 1105 |
| Hymenoptera | *Lasius fuliginosus* | 950 | 1333 |
| Hymenoptera | *Camponotus herculeanus* | 1320 | 1325 |
| Hymenoptera | *Formica rufa* | 1320 | 1325 |
| Aves | *Corvus corone* | 750 | 1406 |
| Aves | *Turdus merula* | 1320 | 1350 |
| Aves | *Tetrao urogallus* | 1150 | 1368 |
| Aves | *Motacilla alba* | 950 | 1368 |
| Aves | *Cyanistes caeruleus* | 1320 | 1142 |
| Aves | *Linaria cannabina* | 700 | 690 |
| Aves | *Fringilla coelebs* | 1320 | 1420 |
| Aves | *Dendrocopos major* | 1320 | 1287 |
| Aves | *Picoides tridactylus* | 1320 | 1406 |
| Aves | *Garrulus glandarius* | 1320 | 1333 |
| Aves | *Spinus spinus* | 1400 | 1420 |
| Aves | *Loxia curvirostra* | 1320 | 1420 |
| Aves | *Sylvia borin* | 750 | 1368 |
| Aves | *Phoenicurus phoenicurus* | 1100 | 1420 |
| Aves | *Motacilla cinerea* | 950 | 1247 |
| Aves | *Pyrrhula pyrrhula* | 1150 | 1328 |
| Aves | *Serinus serinus* | 700 | 1241 |
| Aves | *Emberiza citrinella* | 800 | 1161 |
| Aves | *Chloris chloris* | 750 | 1154 |
| Aves | *Accipiter gentilis* | 1150 | 847 |
| Aves | *Tetrastes bonasia* | 1320 | 1319 |
| Aves | *Lophophanes cristatus* | 1320 | 1396 |
| Aves | *Phoenicurus ochruros* | 1150 | 1352 |
| Aves | *Prunella modularis* | 800 | 1420 |
| Aves | *Columba oenas* | 1320 | 1157 |
| Aves | *Coccothraustes coccothraustes* | 750 | 1333 |
| Aves | *Dendrocopos minor* | 1320 | 1323 |
| Aves | *Parus major* | 1150 | 1245 |
| Aves | *Corvus corax* | 600 | 1350 |
| Aves | *Cuculus canorus* | 1320 | 1333 |
| Aves | *Buteo buteo* | 1050 | 1406 |
| Aves | *Apus apus* | 750 | 1296 |
| Aves | *Delichon urbicum* | 750 | 1161 |
| Aves | *Turdus viscivorus* | 1320 | 1352 |
| Aves | *Sylvia atricapilla* | 1050 | 1420 |
| Aves | *Hirundo rustica* | 750 | 1333 |
| Aves | *Aegolius funereus* | 1050 | 1108 |
| Aves | *Turdus torquatus* | 1400 | 1420 |
| Aves | *Columba palumbus* | 800 | 1312 |
| Aves | *Erithacus rubecula* | 1150 | 1396 |
| Aves | *Aegithalos caudatus* | 750 | 1177 |
| Aves | *Dryocopus martius* | 1320 | 1226 |
| Aves | *Turdus philomelos* | 1320 | 1396 |
| Aves | *Regulus ignicapilla* | 1400 | 1328 |
| Aves | *Glaucidium passerinum* | 1050 | 1304 |
| Aves | *Nucifraga caryocatactes* | 1150 | 1090 |
| Aves | *Periparus ater* | 1320 | 1406 |
| Aves | *Falco tinnunculus* | 1050 | 1177 |
| Aves | *Turdus pilaris* | 1050 | 794 |
| Aves | *Certhia familiaris* | 1320 | 1406 |
| Aves | *Strix aluco* | 1150 | 778 |
| Aves | *Scolopax rusticola* | 1320 | 855 |
| Aves | *Cinclus cinclus* | 1100 | 843 |
| Aves | *Poecile montanus* | 1150 | 1159 |
| Aves | *Anthus pratensis* | 700 | 1340 |
| Aves | *Regulus regulus* | 1400 | 1420 |
| Aves | *Troglodytes troglodytes* | 1320 | 1406 |
